# Advances in targeted therapy for esophageal cancer

**DOI:** 10.1038/s41392-020-00323-3

**Published:** 2020-10-07

**Authors:** Yan-Ming Yang, Pan Hong, Wen Wen Xu, Qing-Yu He, Bin Li

**Affiliations:** 1 MOE Key Laboratory of Tumor Molecular Biology and Key Laboratory of Functional Protein Research of Guangdong Higher Education Institutes, Institute of Life and Health Engineering, Jinan University, Guangzhou, China; 2 MOE Key Laboratory of Tumor Molecular Biology and Guangdong Provincial Key Laboratory of Bioengineering Medicine, National Engineering Research Center of Genetic Medicine, Institute of Biomedicine, College of Life Science and Technology, Jinan University, Guangzhou, China

**Keywords:** Tumour biomarkers, Oncogenes, Drug development, Gastrointestinal cancer

## Abstract

Esophageal cancer (EC) is one of the most lethal cancers in the world, and its morbidity and mortality rates rank among the top ten in China. Currently, surgical resection, radiotherapy and chemotherapy are the primary clinical treatments for esophageal cancer. However, outcomes are still unsatisfactory due to the limited efficacy and severe adverse effects of conventional treatments. As a new type of approach, targeted therapies have been confirmed to play an important role in the treatment of esophageal cancer; these include cetuximab and bevacizumab, which target epidermal growth factor receptor (EGFR) and vascular endothelial growth factor (VEGF), respectively. In addition, other drugs targeting surface antigens and signaling pathways or acting on immune checkpoints have been continuously developed. For example, trastuzumab, a monoclonal antibody targeting human epidermal growth factor receptor 2 (HER-2), has been approved by the Food and Drug Administration (FDA) as a first-line treatment of HER-2-positive cancer. Moreover, the PD-L1 inhibitor pembrolizumab has been approved as a highly efficient drug for patients with PD-L1-positive or advanced esophageal squamous cell carcinoma (ESCC). These novel drugs can be used alone or in combination with other treatment strategies to further improve the treatment efficacy and prognosis of cancer patients. Nevertheless, adverse events, optimal dosages and effective combinations still need further investigation. In this review, we expound an outline of the latest advances in targeted therapies of esophageal cancer and the mechanisms of relevant drugs, discuss their efficacy and safety, and provide a clinical rationale for precision medicine in esophageal cancer.

## Introduction

Esophageal cancer (EC), including esophageal squamous cell carcinoma (ESCC) and esophageal adenocarcinoma (EAC), is one of the most lethal cancers in the world. ESCC, the predominant type in Southeast Asia and Africa, is a highly lethal cancer that occurs in the middle or upper part of the esophagus. ESCC usually originates from the lining of esophageal squamous epithelium. When the esophageal mucosa is exposed to carcinogens or mechanically damaged, the epithelial cells will abnormally proliferate, and eventually develop into invasive cancer.^1,2^ EAC, the predominant type in Europe and North America, usually occurs in the lower middle of the esophagus and originates from glandular cells near the stomach.^2,3^ The pathogenesis of EAC involves the abnormal proliferation of esophageal epithelial cells triggered by gastroesophageal reflux disease (GERD), which eventually develops into invasive adenocarcinoma.^2,4^ Although ranking eighth in terms of incidence, EC ranks sixth among all cancers in mortality.^5^ In the past three decades, unlike traditional therapies, newly developed treatments in EC have had no profound clinical impact, resulting in minimal improvements in survival.^6^ Despite these circumstances, scientists believe that EC therapies remain promising. In the past decade, the Food and Drug Administration (FDA) approved three kinds of targeted therapy for EC treatment, and several drugs are awaiting approval.^7^ To date, scientists have developed multiple monoclonal antibodies and tyrosine kinase inhibitors (TKIs), which can be utilized alone or in combination with traditional therapies to improve the prognosis of patients with ESCC. Here, we have summarized recent advances in targeted therapies against oncogenes (EGFR, HER-2, VEGFR, c-Met, etc.), epigenetic lesions, immune components, and the microbial ecosystem in EC.

## Targeting key signaling pathways

### Targeting the EGFR pathway

The epidermal growth factor receptor (EGFR) is a member of the ErbB family of receptor tyrosine kinases (RTKs). Binding of its ligand epidermal growth factor (EGF) triggers homodimerization or heterodimerization and subsequent autophosphorylation of the receptor. This in turn activates downstream effectors, such as the RAS-RAF-MEK-ERK-MAPK and PI3K-AKT-mTOR pathways, which play roles in cell proliferation, differentiation, and survival.^8,9^ On the other hand, abnormal activation of EGFR, such as overexpression of receptors and heterodimerization of ligand-dependent receptors, is also relevant to carcinogenesis. As a result, in the development of chemotherapy and molecular-targeted therapy of EC, EGFR is a vital candidate. Recently, many related drugs have been developed to inhibit EGFR signaling, thereby effectively treating EC. For example, monoclonal antibodies, such as cetuximab and nimotuzumab, recognize the extracellular region of EGFR to block the binding of EGF to the receptor, subsequently inhibiting the carcinogenesis of EC. In addition, erlotinib and gefitinib, small-molecule inhibitors that act on the intracellular region of the receptor, can inhibit intracellular tyrosine kinase activity and thus suppress the occurrence and development of cancer. For the EGFR targeted therapy in esophageal cancer, drug resistance due to mutations in EGFR-related genes must be considered. For example, the activation of the JAK/STAT pathway contributes to drug resistance of gefitinib, and cucurbitacin B, an inhibitor of JAK/STAT signaling, can be used in combination with gefitinib to overcome chemoresistance and enhance treatment efficacy. Refer to this kind of strategy, we can combine targeted drugs with other treatment methods as much as possible to develop a combined plan; or we can use related methods, such as gene silencing, to inhibit pathways related to tumor resistance.^10,11^ In addition, another new technology for EGFR targeted therapy is to package and deliver targeted drugs to specific locations in cells through related carriers, such as liposome nanoparticles, to achieve precise positioning of the therapeutic target, and finally improve the effect of treatment.^12^

#### Cetuximab

Cetuximab, a monoclonal antibody against EGFR, specifically targets EGFR to inhibit its activation, which impedes cancer progression. Many studies have indicated that cetuximab is useful in treating EC, particularly when combined with other therapies. T. Ruhstaller and other scientists reported that the addition of cetuximab to other treatments (such as radiotherapy, chemotherapy, and surgery) as an adjunct therapy can significantly mitigate the development of regional ESCC.^13^ In addition, cetuximab improves progression-free survival (PFS) and overall survival (OS) in patients with resectable ESCC without increasing toxicity or postoperative morbidity. According to Huang’s report, for patients with ESCC with high EGFR expression, cetuximab could not only improve survival but also reduce the recurrence and metastatic potential of the tumor.^14^ Combining cetuximab with chemotherapy drugs is a novel strategy for esophageal cancer therapy. Yar Saglam et al. revealed that the combination of cetuximab and cucurbitacin B can significantly inhibit growth and migration of colorectal cancer cells. Similarly, whether this combination could improve the utilization of targeted agents in esophageal cancer warrants investigation. On the other hand, it is necessary to consider toxicity caused by combined therapy and reduce the occurrence of adverse reactions.^10^ However, cetuximab may not benefit cancer patients with low EGFR expression. Therefore, further research on the clinical implications of cetuximab should focus on patients with high EGFR expression.

#### Nimotuzumab

Nimotuzumab, a fully recombinant, humanized monoclonal antibody, is the first monoclonal antibody in China used to treat malignant tumors. In the treatment of ESCC, EGFR is a potential therapeutic target. Compared with cetuximab, nimotuzumab causes less toxicity and has a lower incidence of rash.^15–17^ In comparison with conventional chemotherapy, nimotuzumab has a better effect in patients with ESCC. Studies have found that nimotuzumab in combination with chemoradiation can improve the treatment efficacy and prognosis of ESCC without toxicity.^18^ Moreover, nimotuzumab combined with chemotherapy can achieve promising clinical results in locally advanced or metastatic ESCC, with no toxicity and good tolerance. Therefore, this combination regimen may become a novel treatment for patients with metastatic ESCC.^18^

#### Gefitinib

Gefitinib is an EGFR inhibitor that can block the downstream signaling of EGFR in cells, thus inhibiting the development and progression of cancer. A large number of studies have revealed that gefitinib can improve the survival of patients with non-small-cell lung cancer and can be used in treating EC, including EAC and ESCC.^19,20^ Petty et al. used FISH to confirm that gefitinib can be beneficial to patients with EAC or ESCC who worsen after chemotherapy and whose PFS was improved; furthermore, rapid and durable responses were observed in a few patients.^21^ Xu et al. followed 41 patients with advanced ESCC who received cisplatin or 5-fluorouracil (5-FU) in combination with gefitinib^22^ and found that gefitinib combined with radiotherapy and chemotherapy actually improved the survival and quality of life of patients with advanced ESCC. Furthermore, the toxic effects of gefitinib combination therapy included only grade 1 to 3 nonserological toxicities. In general, gefitinib is effective in treating ESCC and improves the survival of patients with advanced ESCC, but its efficacy with other anti-EGFR therapies needs to be further explored.^22^

#### Icotinib

Icotinib is a potent, highly selective EGFR inhibitor.^23^ It is the first targeted small-molecule drug developed by scientists in China for use as a novel type of anticancer treatment.^24^ Icotinib is effective in the treatment of ESCC in patients with high EGFR expression. Huang et al. observed EGFR overexpression or EGFR gene amplification in patients with advanced ESCC. After receiving oral icotinib (250 mg, 3 times daily), these patients showed increased median PFS and OS values. Therefore, this observation indicates that the activity of icotinib is favorable in patients with advanced treated ESCC with EGFR overexpression or gene amplification.^25^ However, the therapeutic effect of icotinib still requires further study.

### Targeting the HER2 pathway

Human epidermal growth factor receptor 2 (HER-2), a member of the EGFR family, is a tyrosine kinase that localizes to cell membrane and conducts extracellular-intracellular signaling to regulate cell growth and differentiation, as well as the development of cancer.^26,27^ At present, HER-2 expression has been detected in various tumors, such as esophageal and gastric cancer, breast cancer, and ovarian cancer.^28–30^ Therefore, HER-2 is a crucial target for molecular-based treatment of EC. Currently, trastuzumab and lapatinib are the two primary therapeutic drugs targeting HER-2.

#### Trastuzumab

Trastuzumab, an antibody-drug conjugate, consists of a humanized antibody against HER-2, a novel enzyme-cleavable linker and a topoisomerase I inhibitor.^31^ In clinical trials, trastuzumab could inhibit the growth of tumors with HER-2 expression.^32^ Doi explored the safety and tolerability of trastuzumab in patients with advanced gastric or gastro-esophageal cancer and found that trastuzumab could ameliorate and control the development of disease in most patients. Even in tumors with low HER-2 expression, trastuzumab also showed antitumor activity. The researchers advised that the optimal dose of trastuzumab as a second-line therapeutic is 5.4 to 6.4 mg/kg.^31^ Although trastuzumab is the standard first-line treatment for HER-2-positive EAC, not every patient responds positively. According to reports, its response rate (RR) ranges from 30% to 60%.^33–35^ Therefore, even though some EAC patients are defined as HER-2 positive, they do not respond to trastuzumab. Combination treatment including trastuzumab and other therapies for EAC needs further research.

#### Lapatinib

Lapatinib, a small-molecule inhibitor of EGFR and HER-2, has been approved to treat HER-2-positive breast cancer.^36^ Guo’s team reported that the combination of lapatinib and paclitaxel had a highly synergistic effect on inhibiting cell proliferation and significantly reduced the invasion and migration of ESCC cells.^37^ Treatment of ESCC cells with lapatinib and paclitaxel markedly increased apoptosis via inhibition of EGFR and HER2 phosphorylation and inactivation of the MAPK and AKT signaling pathways, suggesting that lapatinib could exert a greater antitumor effect when combined with paclitaxel compared with that of monotherapy. In a randomized phase III clinical trial, the patients with advanced esophageal adenocarcinoma received the combination therapy with lapatinib and CapeOx. The results revealed that median OS in the combination therapy group and placebo group was 12.2 months and 10.5 months, respectively, whereas the progression-free survival in combination therapy and placebo groups was 6.0 months and 5.4 months, respectively. Besides, the response rate in the combination therapy group was significantly higher than the control group: 53% compared with 39%, suggesting that the addition of lapatinib shows an obvious therapeutic effect in the treatment of esophageal adenocarcinoma.^38^

### Targeting the VEGF/VEGFR pathway

Tumor angiogenesis involves many complex processes, including interactions between regulators and effectors. Vascular endothelial growth factors (VEGFs), including VEGF-A, VEGF-B, VEGF-C, VEGF-D, and PlGF, are regulators of angiogenesis and play an important role in the proliferation and angiogenesis of vascular endothelial cells.^39^ Vascular endothelial growth factor receptor (VEGFR) is a specific receptor for VEGFs. VEGFs are secreted by tumors or stromal cells and function in an autocrine or paracrine manner by binding to VEGFRs (VEGFR1, VEGFR2, and VEGFR3).^40^ The VEGF/VEGFR interaction can trigger a variety of signaling pathways, such as the extracellular regulated protein kinases 1/2 (ERK1/2) and phosphatidylinositol 3-kinase/protein kinase B (PI3K/AKT) pathways, leading to elevated cell proliferation, migration, and survival.^41,42^ VEGF expression is significantly related to the progression and prognosis of EC;^43–45^ therefore, the VEGF/VEGFR signaling pathway is a potent target for the treatment of EC.

#### Bevacizumab

Bevacizumab is a monoclonal antibody that targets VEGF-A. It can increase vascular permeability and inhibit tumor growth by preventing VEGF-A from binding to VEGFR2. In a phase II-III clinical trial, patients with EAC received the combination of bevacizumab and chemotherapy, while the patients in the control group received only chemotherapy. Compared with the control group, the experimental group had a lower 3-year OS rate (48.1% vs. 50.3%) and were more likely to manifest wound healing complications. Among patients undergoing esophageal gastrectomy, the postoperative anastomotic fistula rate was higher in the group treated with chemotherapy plus bevacizumab. Because of the adverse effects of bevacizumab on wound healing, it should not be used for routine treatment of esophagogastric adenocarcinoma during surgery.^46^

#### Ramucirumab

Ramucirumab is a new type of human immunoglobulin G (IgG) 1 monoclonal antibody that can specifically inhibit VEGFR2 and block its interaction with ligands, further inhibiting angiogenesis and inducing tumor cell apoptosis.^47,48^

Ramucirumab has been studied in EC and other related cancers (Table 1). A phase II clinical study of ramucirumab combined with oxaliplatin (FOLFOX) for advanced esophageal or gastric/gastroesophageal junction (GEJ) adenocarcinoma showed that adding ramucirumab to FOLFOX did not significantly improve the median PFS. However, the PFS at three months was different, and the disease control rate (DCR) was considerably improved.^49^ The RAINBOW trial was used to evaluate the efficacy and safety of paclitaxel in combination with ramucirumab in patients with advanced esophagus or gastric/GEJ adenocarcinoma who were treated with chemotherapy.^50^ The results showed that OS was significantly longer in the combination treatment group than in the placebo plus paclitaxel group (9.6 and 7.4 months, respectively).

**Table 1:** Summary of various clinical trials of ramucirumab

Proposal	Disease stage	Treament	Adverse events	Results	Trial
Ramucirumab	Advanced or metastatic gastroesophageal cancers	1st line monotherapy	Hypertension	Median OS: 5.2 months Median PFS: 5.1 months	Wilke H et al. ^50^
Ramucirumab+Paclitaxel vs. Paclitaxel	Metastatic esophagogastric junction adenocarcinoma	1st line in chemotherapy	Hypertension Leukopenia Neutropenia	Median OS: 9.6 months vs 7.4 months Median PFS: 4.4 months vs 2.86 months	Tabernero J et al.^112^
Ramucirumab+Fluoropyrimidine+Cisplatin vs. Placebo	Gastroesophageal adenocarcinoma	1st line in chemotherapy	Anemia Leukopenia Neutropenia	Median OS: 11.2 months vs 10.7 months Median PFS:5.7 months vs 5.4 months	Fuchs CS et al.^113^
Ramucirumab (8 mg/kg every 2 week)	Advanced or metastatic gastroesophageal cancers	2nd line therapy	Diarrhea Decreased appetite	12-weeks PFS rate: 23.8% [90%(Cl):12.4-37.2]	Yamaguchi et al.^114^
Ramucirumab+Oxaliplatin vs. Placebo+Oxaliplatin	Advanced esophageal, GEJ, or gastric adenocarcinoma	2nd line therapy	Peripheral sensory neuropathy	The group with RAM+SOX did not show an improvement in PFS compared with PBO+SOX	Kei Muro et al.^115^

The side effects of ramucirumab include hypertension, thromboembolism, rash, diarrhea, and myelosuppression. In a phase III clinical trial in a first-line setting, ramucirumab was administered to patients with advanced gastric/GEJ adenocarcinoma after chemotherapy with drugs containing platinum or fluoropyrimidine. The results showed that the OS of patients who received ramucirumab was 5.2 months, while that of the placebo group was 3.8 months. The incidence of hypertension in the ramucirumab group was higher than that in the placebo group (16% and 8%, respectively), whereas the incidences of other adverse reactions were similar between the two groups (94% and 88%, respectively)^51^. In summary, ramucirumab is beneficial for the survival of patients with advanced gastric/GEJ adenocarcinoma after first-line chemotherapy

#### Endostar

Developed by Chinese scientists, Endostar is a novel recombinant human endostatin consisting of nine amino acid residues linked with the N-terminus of the endostatin protein. The C-terminus contains a proteolytic fragment from type XVIII collagen, which is normally found in blood vessels and epithelial basement membranes, Endostar has been confirmed to be effective in suppressing angiogenesis and tumor growth.^52^ In 2005, Endostar was approved by the China Food and Drug Administration (CFDA) for use with cisplatin or other chemotherapy drugs to treat small cell lung cancer.^53^ Endostar inhibits the migration of vascular endothelial cells and increases the activity of vascular endothelial cell growth inhibitor (VEGI), subsequently blocking VEGF activity and subsequent tumor growth.

Chang et al. studied the effect of Endostar in combination with chemotherapy on the ESCC cell line Eca-109 in mice and found that the combination of Endostar and chemotherapy reduced the tumor weight compared with that of either treatment alone.^54^ In addition, the proliferation of tumor xenografts also declined, suggesting that combining Endostar and chemotherapy inhibit ESCC cells. In a clinical trial, Xu et al. observed a 71-year-old female with ESCC who received chemotherapy plus Endostar (3 mg; days 1–14; intravenously) with sequential SBRT (stereotactic body radiation therapy, 3300 cGy in 10 fractions). After treatment, the patient’s symptoms disappeared, and her PFS was >8 months, indicating that Endostar is a good option for treating ESCC when combined with chemotherapy and radiotherapy.^55^

#### Sunitinib

Sunitinib is a small-molecule inhibitor against RTKs that can selectively target VEGFR1, VEGFR2, and VEGFR3 to block their activities, subsequently inhibiting tumor growth.^56^ Phase II clinical trials of sunitinib as an adjuvant for FOLFIRI (irinotecan + fluorouracil + calcium folic acid) showed that sunitinib combined with FOLFIRI did not improve PFS in patients with chemotherapy-resistant advanced gastric or GEJ cancer. However, a tendency for prolonged OS was noted.^57^

#### Sorafenib

Sorafenib is a multikinase inhibitor with a similar mechanism to sunitinib and can selectively inhibit the biological activity of VEGFR to suppress the development of tumors. A study of sorafenib combined with DP (docetaxel + cisplatin) in the treatment of advanced gastric or GEJ cancer showed that the median PFS was 5.8 months, and the median OS was 13.6 months. The effect of this combination regimen was acceptable, with neutropenia as the only adverse effect observed.^58^ Another phase II clinical trial reported that sorafenib inhibited the progression of advanced EAC and advanced GEJ cancer and prolonged PFS.^59^

#### Apatinib

As a small-molecule inhibitor, apatinib was independently developed in China and has been shown to suppress the activity of VEGFR and HER2. A clinical trial indicated that apatinib could be used as a second-line or third-line treatment for advanced ESCC.^60^ A phase II trial in a first-line setting tested apatinib, the PD-1 inhibitor camrelizumab, and chemotherapy in ESCC. Although patient survival data are still being evaluated, the combination of drugs has shown promising efficacy.^61^ Studies of apatinib combined with docetaxel for advanced ESCC have revealed that this combination therapy can be used as second-line or further treatment for patients with advanced ESCC.^62^

#### Anlotinib

Anlotinib is a small-molecule inhibitor that can act on multiple targets. For example, this drug can selectively inhibit VEGFR and contribute to the inhibition of tumor growth and metastasis. Phase II clinical trials for advanced ESCC have shown that anlotinib significantly improved PFS and the DCR of ESCC.^63^ Based on this study, anlotinib monotherapy was included in the 2019 version of the Chinese Society of Clinical Oncology (CSCO)-EC guidelines as a second-line and beyond treatment for ESCC.

### Targeting the HGF/c-Met pathway

Hepatocyte growth factor receptor (c-Met) is a receptor for hepatocyte growth factor (HGF). MET is mainly expressed in epithelial cells, while HGF is produced and secreted by surrounding mesenchymal cells. This ligand/receptor-mediated interaction between the epithelium and matrix is significant under physiological conditions and plays an important role in the regulation of tumor cell growth, invasion, metastasis, and angiogenesis. Many tumors constitutively express MET and HGF/SF (scatter factor) to escape multiple regulatory mechanisms.^64^ c-Met is overexpressed in EAC, which is related to the poor prognosis in EAC treatment.^65^ Yan et al. demonstrated that the protein expression of c-Met was higher in EC tissues than in adjacent tissues, and high c-Met expression was related to clinical stage, depth of invasion, and lymph node metastasis.^66^ Therefore, quite a few drugs targeting the c-Met/HGF pathway are also in development and function by either preventing the binding of HGF and c-Met or directly targeting c-Met to subsequently inhibit the c-Met/HGF signaling pathway.

#### Rilotumumab (AMG-102)

Rilotumumab is a humanized monoclonal antibody that targets HGF to inhibit its interaction with c-Met. Phase III clinical trials evaluating rilotumumab in combination with epirubicin, cisplatin, and capecitabine for MET-positive gastric/GEJ adenocarcinoma have shown that rilotumumab cannot effectively treat gastric/GEJ adenocarcinoma with MET-positive expression.^67^ Rilotumumab has still not earned a place as a targeted therapy ESCC or EAC.

#### Obinutuzumab

Obinutuzumab is a fully humanized anti-MET antibody that inhibits the binding of MET to HGF. The results of phase II and phase III clinical trials of obinutuzumab combined with FOLFOX chemotherapy in gastric/GEJ adenocarcinoma have shown that obinutuzumab cannot improve PFS in MET-positive patients. In addition, to date, phase III trials have failed to show any survival benefits.^68,69^

#### Other c-MET inhibitors

Crizotinib is a dual-target TKI against ALK and c-MET. It has been reported to exert a specific influence on EC with c-Met amplification, but large-scale clinical studies have not verified its effects. AMG337, a small-molecule inhibitor of MET, can effectively inhibit c-Met/HGF binding.^70^ At present, completed phase I and II clinical trials have verified that AMG337 showed antitumor activity in patients with EAC with MET amplification.^71,72^ Other small-molecule TKIs, such as telatinib and vorinostat, are in clinical trials at various phases, and more information is needed.

### Targeting the mTOR-related pathway

Mammalian target of rapamycin (mTOR) is a serine/threonine protein kinase that is responsible for regulating protein synthesis, ribosomal protein translation, and cap-dependent translation. Dysregulation of mTOR signaling plays a crucial role in tumorigenesis, angiogenesis, cell growth, and metastasis.^73^ Elevated levels of p-mTOR are related to poor prognosis in ESCC, which lends itself as a therapeutic target for ESCC.^74^ Everolimus is an mTOR inhibitor with an excellent antitumor effect. In vitro studies have shown that in most ESCC cells, the mTOR pathway is abnormally activated, and everolimus alone or in combination with cisplatin exerted a therapeutic influence on these cells.^75^

### Targeted therapy for other pathways

Fibroblast growth factor receptor-2 (FGFR2), a member of the RTK family, regulates cell proliferation and angiogenesis and is amplified in 4–10% of esophageal and gastric cancers.^76^ Phosphatidylinositol 3-kinase (PI3K) is a lipid kinase that can regulate the growth, proliferation, and migration of tumor cells. According to an analysis of ESCC patients from the TCGA database, PI3K has abnormal expression in ESCC and can affect survival in patients with ESCC.^76^ Both these proteins are expected to be potential therapeutic targets for ESCC.

### Discussion

**Fig. 1:**
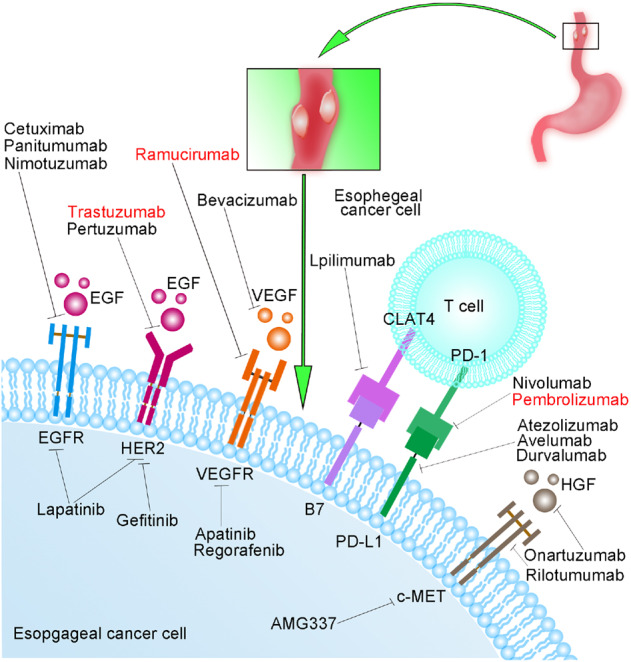
The most studied targets and related drugs in esophageal cancer. The drugs in red text have been approved by the FDA for the treatment of esophageal cancer, and the remaining drugs in black text are still in different stages of research. EGF epidermal growth factor, EGFR epidermal growth factor receptor, HER2 human epidermal growth factor receptor 2, VEGF vascular endothelial growth factor, VEGFR vascular endothelial growth factor receptor, CTLA-4 cytotoxic T-lymphocyte-associated antigen 4, PD-1 programmed death receptor 1, PD-L1 programmed death-ligand 1, HGF hepatocyte growth factor, c-MET c-mesenchymal-epithelial transition

**Fig. 2:**
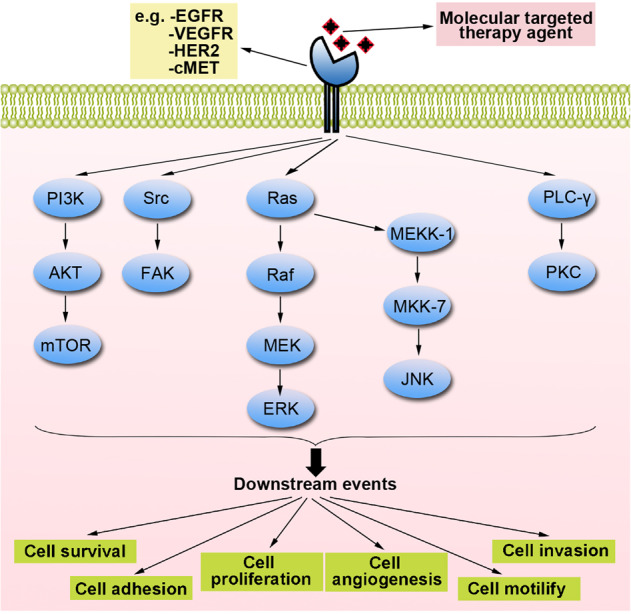
EGFR-related, VEGFR-related, HER2-related, and c-MET-related signaling pathways. These receptors on the cell membrane are activated by binding to the corresponding ligands (therapeutic drugs), which lead to autophosphorylation of tyrosine residues. The signaling pathways include the PI3K/AKT/mTOR, Src/FAK, Ras/Raf/MEK/ERK, Ras/MEKK-1/MKK-7/JNK, and PLC-γ/PKC pathways. By regulating these pathways, targeted drugs can ultimately affect the survival, adhesion, proliferation, angiogenesis, movement, and invasion of cancer cells

In summary, we have highlighted the most studied and important targets and their related drugs in EC (Fig. 1), which are usually used in combination with chemotherapy and/or radiotherapy and ultimately improve the prognosis of patients. Trastuzumab and ramucirumab have been approved by the FDA for EC therapy. Some targeted drugs are still in the preclinical stage, and their efficacies are currently being tested. What these drugs have in common is that they act on a critical target receptor on the membrane of EC cells and ultimately inhibit the relevant downstream activities to achieve therapeutic effects. As shown in Fig. 2, receptors such as EGFR, VEGER, HER2, and c-MET are regarded as significant targets for ESCC and EAC therapy. Different targeted drugs will bind specifically to their corresponding receptor and affect different downstream signaling pathways, such as the Ras/Raf/MEK/ERK pathway and PI3K/AKT/mTOR pathways. Ultimately, these drugs affect the activity of cancer cells, such as cell adhesion, proliferation and invasion. However, mutations in related genes within these pathways can lead to drug resistance. For example, mutations of the Ras and Raf genes may result in resistance to EGFR antibody, and mutations in genes associated with the PI3K/AKT pathway are related to resistance to HER2 antibody.^77^

At present, although EGFR, VEGFR, HER2, and c-Met have been extensively studied, the specific mechanisms involved in their activities are poorly defined, which is a challenge for improving the effectiveness of targeted therapies for EC. Therefore, scientists must continue to conduct in-depth studies to improve the efficacy of drugs and develop more effective targeted drugs.

## Epigenetic-targeted therapy

### DNA methyltransferase inhibitors

Patients with EC are likely to sustain hypermethylation of the CpG islands at the promoters of tumor suppressor genes. DNA methyltransferases regulate DNA methylation and genes are often highly expressed in tumor tissues; thus, they could be useful targets for tumor therapy. 5-Azacitidine and decitabine are a nucleoside analog and its deoxy derivative, respectively. These molecules can be metabolized in vivo to form deoxynucleoside triphosphate, which can replace cytosine during DNA replication and block the action of DNA methyltransferases. Patients with advanced esophageal/gastric adenocarcinoma (EGC) were treated with neoadjuvant EOX (epirubicin, oxaliplatin, and capecitabine) chemotherapy and were given a 5-phase subcutaneous injection of azacitidine before chemotherapy in a phase I clinical study. The results demonstrated that 5-azacitidine can enhance the efficacy of chemotherapy.^78^

### Histone modification inhibitors

Histone modification inhibitors include histone deacetylase inhibitors (HDACis), histone methyltransferase inhibitors (HMTis), histone demethylase inhibitors (HDMis), and histone acetyltransferase inhibitors (HATis). Some HDACis, including vorinostat, romidepsin, belinostat, and panobinostat, have been approved by the FDA and are primarily used for the treatment of lymphoma and myeloma.^79,80^ At present, there are limited studies on the effects of histone modification inhibitors on EC. Furthermore, there are no clinical studies available on this topic, although some work has been performed in vitro. The combination of an HDACi and 5-azacytidine can selectively inhibit ESCC and EAC cells, suggesting the feasibility of these inhibitors in the targeted treatment of ESCC and EAC.^81^ LPE-1 inhibits the growth and migration of ESCC cells by targeting LSD1, demonstrating that LSD1 may be a potential therapeutic target for ESCC.^82^ Histone modification plays a pivotal role in the occurrence and progression of EC, and relevant research on its targets will have broad prospects.

### Noncoding RNA (ncRNA) inhibitors

Antisense oligonucleotides (ASOs), miRNA sponges, and small interfering RNAs (siRNAs) can inhibit the expression of carcinogenic ncRNAs. Especially for siRNA, it has more advantages than small molecule inhibitors and monoclonal antibody drugs in the targeted therapy of cancer. Because siRNA binds to mRNA through pairing, thereby inhibiting the expression of genes related to cancer growth. Compared with chemical drugs that need to recognize the complex spatial conformation of proteins, the use of siRNA to inhibit the growth of cancer cells is more precise and effective.^83^ Development of a suitable carrier that delivers siRNA into cells is urgently needed to achieve precise positioning and regulation of target genes by siRNA. Moreover, analogs of oligonucleotides and pharmaceutical preparations synthesized in vitro can restore the expression of ncRNAs with tumor suppressor functions. Strategies targeting ncRNAs have not been tested clinically, but they will be an important direction for future studies of targeted treatments of EC.

## Immunotherapy

In addition to targeting intrinsic signaling in cancer cells, immunotherapy, the strategy to enhance the efficacy and specificity of the immune cells to suppress cancer progression, is a hot research area in cancer therapy. The process of cellular immunity can be divided into several steps. Firstly, the receptor on the surface of the T lymphocyte membrane specifically binds to the major histocompatibility complex (MHC) on the surface of antigen presenting cell (APC). Then, the combination of T cells and MHC will trigger further activation, proliferation, and differentiation. Finally, activated T cells will exert immune effects. This series of signal pathways play a vital role in the process of tumor immunity.^84^ Immunotherapy has also been well developed in the treatment of esophageal cancer, such as immune checkpoint inhibitors, which can bind to protein receptors on the surface of T cells or tumor cells to prevent tumor cells from immune escape, as a result, the immune response proceeds normally. Increasing data from phase III clinical trials in different cancers, such as melanoma, non-small cell lung cancer and colorectal cancer, showed satisfactory effects of immune checkpoint inhibitors on growth, migration, invasion and other cancer hallmarks, providinge rationale for targeting immune checkpoint in esophageal cancer.

### PD-1/PD-L1-targeted therapy

The programmed cell death protein 1 (PD-1) pathway is considered an important inhibitory mechanism that modulates T cell failure. One ligand of PD-1, programmed cell death-ligand 1 (PD-L1), has been shown to be expressed in a variety of cancer cells, confirming that cancer cells can escape from the killing effect of the immune system.^85,86^

**Fig. 3:**
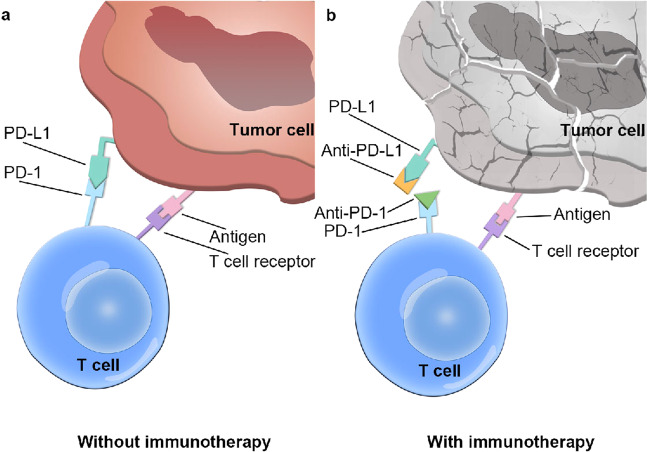
Mechanism of anti-programmed death 1 (PD-1) receptor-programmed and anti-programmed death-ligand 1 (PD-L1) inhibitor-mediated cancer immunotherapy. **a** When PD-1 binds to PD-L1, the immune effect of T cells is suppressed, leading to immune escape. **b** The therapeutic antibodies (anti-PD-1 and anti-PD-L1) bind to PD-1 and PD-L1, respectively, blocking the interaction of PD-1 and PD-L1 and releasing the immunosuppression of T cells. Subsequently, cancer cell lysis will occur

Therefore, PD-L1 inhibitors are considered effective targeted drugs to address cancer cell evasion of T cells. Common PD-L1 inhibitors such as pembrolizumab can bind to PD-L1 on tumor cells, which prevents PD-L1 from binding to PD-1 on T cells and subsequently halting T cell inhibition, which enables T cells to exert a killing effect on cancer cells (Fig. 3). After the pembrolizumab monotherapy for ESCC and EAC patients in a phase II KEYNOTE-180 clinical study, the overall remission rate was 10%, and the mOS was 5.8 months, and the objective response rates (ORR) of ESCC and EAC patients were 14.3% and 5.2%, respectively. Besides, the ORR of PD-L1-positive patients were higher than the PD-L1 negative patients, which were 13.8% and 6.3%, respectively,^4,87^ suggesting that pembrolizumab treatment is effective for PD-L1-positive ESCC patients. Therefore, the FDA approved pembrolizumab as a second-line treatment option for PD-L1-positive advanced ESCC patients, although the adverse events, such as nausea, vomiting, hypertension, caused by pembrolizumab treatment still need to be further explored. In addition, we can try to explore the therapeutic effect of combining pembrolizumab with chemotherapy drugs, such as paclitaxel and oxaliplatin, in ESCC or EAC, and further study the combined treatment of pembrolizumab. Camrelizumab is a humanized anti-PD-1 monoclonal antibody independently developed in China. A phase III clinical trial of camrelizumab in the treatment of ESCC was successfully completed and showed that camrelizumab monotherapy could significantly prolong the survival of patients with advanced or metastatic ESCC who failed to respond to first-line chemotherapy, which meant that camrelizumab is likely to be approved for the treatment of ESCC. The treatment effects and associated prognosis of combination therapies comprising PD-1/PD-L1 inhibitors and radiotherapy and/or chemotherapy in patients with ESCC need to be assessed in further studies.

### CTLA4-targeted therapy

**Fig. 4:**
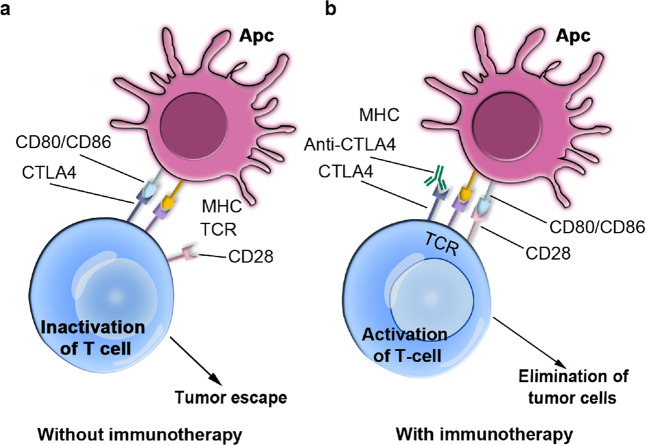
The mechanism of CTLA-4 immune checkpoint blockade by therapeutic antibodies employed in cancer immunotherapy. **a** CTLA-4 on T cells competes with CD28 to bind to B7 ligands on APCs (antigen-presenting cells). Once CTLA-4 binds to B7, the activity of T cells will be suppressed, and immune escape can occur. **b** After anti-CTLA-4 binds CTLA-4, CD28 will bind to B7, which then activates the T cell and ultimately leads to the death of the cancer cells

Cytotoxic T-lymphocyte associated protein 4 (CTLA4) is a negative regulator of T cell expression, whose upregulation can decrease the expression of interleukin-2 (IL-2). CTLA4 can arrest T cells in G1 phase of the cell cycle, reduce the specific immune function of the body and promote the immune escape of cancer cells.^88^ Some studies have proven that CTLA4 can serve as an immune-based target for cancer treatment.^89^ Currently available drugs that target CTLA4 include ipilimumab and tremelimumab (Fig. 4).^90,91^

Ipilimumab is a monoclonal antibody that can effectively block CTLA4 and has been widely used in the treatment of melanoma, with great success.^92^ However, there has been no clinical evidence for the application of ipilimumab in the treatment of EC. The phase I/II CheckMate-032 trial (NCT01928394) was conducted to study the efficacy and safety of the combination of ipilimumab either alone or in combination with nivolumab in the treatment of solid tumors of EC. Janjigian et al. reported the effects of ipilimumab on gastroesophageal cancer and found that the OS of gastroesophageal cancer patients who received the combination of ipilimumab and nivolumab was 6.9 months, and no adverse reactions were reported; this was consistent with previous relevant reports.^93–97^ The next step in this process is a phase III trial in patients.

Tremelimumab (CP-675206, Pfizer) is a fully humanized monoclonal antibody against CTLA4. It was developed to mimic IgG2, which could minimize complement activation and reduce the risk of a cytokine storm. In a phase II study, the objective remission rate of tremelimumab as a second-line treatment for metastatic EAC was only 5%. However, CT scans showed that tremelimumab was beneficial in controlling disease in a small number of patients.^98^ Some studies have also found that tremelimumab could cause adverse effects such as pruritus, skin rash, eosinophilia, diarrhea and fatigue. In addition, one study showed that CTLA-4 blockade combined with antigen-targeted therapy may be a possible treatment strategy for EC, but this requires further study.^99^

### TIM-3-targeted therapy

T cell immunoglobulin domain and mucin domain-3 (TIM-3) is part of an immune checkpoint and has important research prospects. Unlike other immune checkpoint molecules, TIM-3 is not expressed in all types of T cells, but rather in CD4+ T helper cells and CD8+ cytotoxic T lymphocytes, and exerts significant inhibitory effects on the immunocompetence of T cells. Upon activation by galectin-9, TIM-3 suppresses effector T cells, decreasing the immune response. In addition, TIM-3 plays a significant role in the immune effects of T cells.^100^ Zhao et al. used immunohistochemistry to detect the expression levels of TIM-3 and PD-1 on CD8+ tumor-infiltrating lymphocytes (TILs) in ESCC and analyzed the relationship between clinicopathological features or clinical outcomes and TIM-3 expression.^101^ They found that positive TIM-3 expression was associated with PD-1 positivity and a high density of CD8+ TILs, which is an essential factor affecting RFS (relapse-free survival) and OS in ESCC. In addition, the combination of TIM-3 and PD-1 expression or CD8+ TIL density can be used to stratify ESCC patients into groups with different prognoses. Nevertheless, the characteristics and prognostic value of TIM-3 in ESCC are still uncertain, and there is no drug targeting TIM-3 for ESCC treatment.

### LAG-3-targeted therapy

Lymphocyte activation gene-3 (LAG-3) has been demonstrated to be an important checkpoint in antitumor immunity. It is expressed on TILs, natural killer (NK) cells, and B cells.^102,103^ Recently, Zhang et al. analyzed the expression of LAG-3, CD4, and CD8 in 287 ESCC tissues by immunohistochemistry and found that high expression of LAG-3 was related to a superior survival rate.^104^ In addition, LAG-3 was an independent predictor of the survival rate, which indicates that LAG-3 can become an immune marker that reflects the prognosis of ESCC. However, there are no reports of drugs targeting LAG-3 in the treatment of EC.

### TIGIT-targeted therapy

T cell Ig and ITIM domain (TIGIT) is a member of the Ig superfamily of immunoglobulins.^105,106^ Its expression can inhibit the immune response of NK cells, which permits tumor cells to evade their effects and leads to immune escape. Therefore, TIGIT is an important and worthy target for immunotherapy of EC, with inhibition of TIGIT as a potential immunotherapy strategy.^107,108^ Anti-TIGIT antibodies can be developed to reduce the inhibitory effect of TIGIT on NK cells. Recently, a variety of anti-TIGIT antibodies, such as tiragolumab and mAb-7, have been studied in clinical research. However, no in vitro research or clinical studies on the application of anti-TIGIT antibodies in EC have been reported, so future work on TIGIT-targeted therapies in EC should be considered.

## Microbial ecosystem-targeted therapy

There is a vital relationship between changes in the microbial ecosystem and the development of esophageal or gastrointestinal tumors.^109^ The gut microbiota in the upper digestive tract can interact with the tumor microenvironment and promote the occurrence and development of EC. Tumors affect the gut microbiota by reducing the number and diversity of species in the esophageal mucosal flora, as well as changing the dominant microflora. *Porphyromonas gingivalis* (PG) is an oral pathogen that is related to the degree of ESCC differentiation and lymph node metastasis, but the mechanism by which these bacteria affects ESCC is not clear.^110^ Therefore, scientists should clarify the relationships between microbial ecosystem components, such as PG, and EC. In addition, further research developing new methods for treating EC by regulating the gut microbiota should be performed so that the relevant characteristics of the microbiota can be used for clinical treatment. This research will have significant meaning for microbial ecosystem targeting in EC.

## Conclusion and outlook

In this review, we have summarized the latest research on targeted therapy drugs for esophageal cancer, including ESCC and EAC, and also focused on several drugs that are most likely to be approved for the clinical treatment (Table 2). There is an urgent need to optimize the clinical use of currently available targeted drugs and develop more novel therapeutic strategies with good efficacy and limited side effects for the treatment of EC. Inhibitors of PD-L1 (pembrolizumab), VEGFR (ramucirumab), and HER-2 (trastuzumab) have been verified to improve survival and prognosis in advanced ESCC and EAC. Compared with chemotherapy, pembrolizumab has been shown to be more effective and to have fewer adverse reactions in the treatment of advanced ESCC and EAC; furthermore, it has been approved for use in the second-line and third-line treatment regimens. In addition, due to the limited efficacy and drug resistance of single-agent therapy, the combination of immunotherapy and radiotherapy or chemotherapy is a direction worth navigating. Studies have shown that the combined therapy can affect the tumor microenvironment, increase the response rate of tumor cells to targeted drugs, and ultimately improve disease control.^111^ Encouragingly, ramucirumab has been approved as a second-line treatment for EAC, while trastuzumab has been approved for first-line treatment in combination with chemotherapy in HER-2-positive patients. The remaining targeted drugs still require substantial additional clinical research. Most articles to date have referred to clinical trial data of EAC, but the primary EC subtype in China is ESCC. Therefore, there is an urgent demand for developing a precise plan for preventing and treating EC in China. More importantly, the survival rate of patients treated with chemotherapy for metastatic EC is still low, and we must develop targeted therapies, especially first-line treatments with practical and long-lasting effects.

**Table 2:** Summary of clinical trials of main targeted agents

Agent	Targets	Results	Cancer type	References
Cetuximab	EGFR	mPFS (Placebo vs. Cetuximab): 2.0 months vs. 2.9 months mOS (Placebo vs. Cetuximab): 3.0 months vs. 5.1 months	EAC	^13^
Nimotuzumab	EGFR	mPFS: 13.9 months mOS: 9 months	ESCC	^18^
Gefitinib	EGFR	mPFS: 2.2 months mOS: 6.1 months	ESCC	^22^
Icotinib	EGFR	mPFS: 1.7 months mOS: 3.73 months	ESCC	^26^
Trastuzumab	HER2	mPFS: 7.8 months mOS: 16 months	EAC	^33^
Lapatinib	HER3	PFS: 6.0 months OS: 12.2 months	ESCC	^38^
Bevacizumab	VEGF/VEGFR	3-year overall survival (chemotherapy alone group vs. bevacizumab group): 48.1% vs. 50.3%	EAC	^46^
Ramucirumab	VEGF/VEGFR	mPFS: 5.1 months mOS: 5.2 months	GEJ	^50^
Endostar and Chemotherapy	VEGF/VEGFR	PFS > 8 months	ESCC	^55^
Sunitinib	VEGF/VEGFR	mPFS (Sunitinib+FOLFIRI vs. Sunitinib+Placebo): 3.5 months vs. 3.3 months	GEJ	^57^
Sorafenib	VEGF/VEGFR	mPFS: 5.8 months mOS: 13.6 months	GEJ	^58^
Apatinib	VEGF/VEGFR	PFS: 3.8 months OS: 6.96 months	ESCC	^60^
Anlotinib	VEGF/VEGFR	mPFS (Placebo vs. Anlotinib):1.4 months vs. 3.0 months Disease control rates (DCR): 38.1%	ESCC	^63^
Pembrolizumab	PD-1/PD-L1	mOS: 5.8 months Overall remission rate (ORR): 14.3% vs. 5.2%	ESCC/EAC	^100^
Camrelizumab	PD-1/PD-L1	PFS: 2.0 months OS: 8.0 months	ESCC	^116^
Ipilimumab and Nivolumab	PD-1/PD-L1	mOS (Ipilimumab&Nivolumab vs. Nivolumab): 6.9 months vs. 5.0 months	EC	^89^

At present, the research on related targeted drugs for the treatment of esophageal cancer is still a hotspot. However, while targeted drugs improve the treatment outcome, the disadvantages of the therapy still need to be solved. For example, not only is the cost of targeted therapy expensive than conventional radio therapy and chemotherapy, but its efficacy is questionable. Targeted therapy can prolong the life of patients, but does not achieve a complete recovery due to drug resistance. Although the combined radio-chemotherapy and targeted therapy exerts better efficacy and lower drug resistance than single-agent therapy, the incidence of adverse events caused by the combination therapy has also increased a lot. Finally, because there are many crossovers between the signal pathways regulated by drugs, it is easy to cause other complications. Moreover, the drug response differs by population, which will increase the cost of treatment and monitoring. Therefore, while developing new targeted drugs for esophageal cancer, all these shortcomings of targeted therapy must be taken into consideration and avoided to imporove treatment outcome. We believe that with the discovery of novel therapeutic targets, more and more effective targeted therapies for esophageal cancer will be developed to overcome the lethal disease.
